# Aqueous‐Containing Precursor Solutions for Efficient Perovskite Solar Cells

**DOI:** 10.1002/advs.201700484

**Published:** 2017-11-10

**Authors:** Dianyi Liu, Christopher J. Traverse, Pei Chen, Mark Elinski, Chenchen Yang, Lili Wang, Margaret Young, Richard R. Lunt

**Affiliations:** ^1^ Department of Chemical Engineering and Materials Science Michigan State University East Lansing Michigan 48824 USA; ^2^ Department of Physics and Astronomy Michigan State University East Lansing Michigan 48824 USA

**Keywords:** air processing, aqueous‐containing precursors, humidity, perovskites, solar cells

## Abstract

Perovskite semiconductors have emerged as competitive candidates for photovoltaic applications due to their exceptional optoelectronic properties. However, the impact of moisture instability on perovskite films is still a key challenge for perovskite devices. While substantial effort is focused on preventing moisture interaction during the fabrication process, it is demonstrated that low moisture sensitivity, enhanced crystallization, and high performance can actually be achieved by exposure to high water content (up to 25 vol%) during fabrication with an aqueous‐containing perovskite precursor. The perovskite solar cells fabricated by this aqueous method show good reproducibility of high efficiency with average power conversion efficiency (PCE) of 18.7% and champion PCE of 20.1% under solar simulation. This study shows that water–perovskite interactions do not necessarily negatively impact the perovskite film preparation process even at the highest efficiencies and that exposure to high contents of water can actually enable humidity tolerance during fabrication in air.

Perovskite solar cells have emerged as competitive solution‐processed candidates for photovoltaic applications due to the demonstration of high power conversion efficiency (PCE), ease of fabrication, and low materials cost.^1^, ^2^, ^3^, ^4^, ^5^, ^6^, ^7^, ^8^, ^9^ The efficiency of perovskite solar cells has rapidly risen from 3.8% to over 22.1% just within the past several years.^10^, ^11^ Despite these many advantages, perovskite solar cells still need to overcome several shortcomings before they can be deployed commercially. The main challenges include the fabrication of large‐area modules, the toxicity of lead from perovskite compounds, and the low stability of halide perovskite materials.^12^, ^13^, ^14^ Key among these challenges is addressing the stability of perovskite films and devices, particularly the low moisture stability.^15^, ^16^

Previous research has pinpointed the impact of environmental moisture and moisture present in precursor solutions as one of the important degradation pathways of lead‐halide perovskite compounds that limit the performance of devices.^15^, ^16^, ^17^ Clegg and Hill showed that H_2_O present in PbI_2_ precursors could significantly influence the performance of sequentially spin‐coated perovskite devices.^18^ Increasing the concentration of water was observed to dramatically reduce device performance and exacerbate the hysteresis of the devices. Thus, high moisture sensitivity of organometallic perovskite materials not only implies the need for strict encapsulation, but also requires an avoidance of moisture during device fabrication. Considering this effect, a variety of approaches have been explored to protect against moisture and hydrolysis from the preparation process of perovskite devices. Substantial effort has been made to improve the stability of perovskites by adapting the recipe of the precursor and designing protective capping layers on top of the perovskite film.^19^, ^20^, ^21^, ^22^, ^23^ To achieve this aim, previous work has traditionally been performed under dry fabrication conditions to protect the perovskite precursor and to prevent moisture from contacting films during various stages of processing—typically by processing in glove boxes with parts per million levels of H_2_O.^1^, ^24^ In addition, compatible anhydrous solvents have to be chosen to obtain highly efficient and reproducible perovskite photovoltaics.^19^, ^22^, ^25^ Thus, developing fabrication procedures and perovskite precursors with low moisture sensitivity is not only crucial for achieving high‐efficiency perovskite devices, but is also important for facilitating the commercialization of perovskite photovoltaic devices and enabling greater tolerance for fabrication in air.

While recent research has sought to explain how moisture induces decomposition of perovskite materials and the degradation of the device performance, only a few studies have tried to investigate how H_2_O directly influences the crystallization process of perovskite films. Indeed, it is even possible that H_2_O can improve the crystallization of perovskite films and improve the performance of perovskite devices. Wu et al. reported an inverted perovskite solar cell fabricated by a sequential deposition method where highly dilute 2 wt% H_2_O (in dimethylformamide (DMF)) added to the precursors resulted in devices with the PCE over 18%.^26^, ^27^ Liao and co‐workers also reported a case of adding dilute H_2_O to the perovskite precursor in a one‐step method, where 2% H_2_O enhanced the crystallization, surface coverage, and stability of perovskite thin films and increased the device PCE from 12.1% to 16.0%.^28^ However, in these previous reports the devices only worked well when a small amount of H_2_O was added in the precursor; when the water concentration was increased to over 2%, cell performance was found to dramatically decrease.^26^, ^28^ Conings et al. introduced larger amounts of water (≤10%) into perovskite precursors and investigated the effect on the device performance.^29^ Due to the formation of pinholes when the water ratio reached 10%, the device open‐circuit voltage (*V*_OC_) and fill factor (FF) degraded, leading to a PCE less than 11%. This work was also carried out in glove box to avoid the other adverse effects from air, which ultimately limits the applications of hydrous perovskite precursors in industrial production. In this work, we report a highly efficient perovskite solar cell prepared by aqueous‐containing precursor with over 20% water content processed in air. The morphology and crystallization of the perovskite film prepared by aqueous‐containing and anhydrous precursor have been systematically investigated, and the PCE of aqueous‐precursor‐based devices is shown to reach 20.1% with improved stability, low moisture sensitivity, and negligible hysteresis.

**Figure** **1** shows photographs of the perovskite precursor solutions with various volume ratios of water in the total solvent system. The freshly prepared solutions are homogeneous and clear, not only for the anhydrous solvent precursor, but also for the water‐containing precursor even at the highest water content of 25 vol%. When storing the precursor overnight, both the anhydrous precursor and 20% water content precursor (H_2_O‐20% precursor) remain clear, whereas the 25% water content precursor (H_2_O‐25% precursor) shows precipitate formation. Although the precipitate can be redissolved via heating, this concentration provides a practical water limit to prevent continuous clustering. As a result, we chose the H_2_O‐20% precursor as the primary point of comparison in this work, while higher concentrations (H_2_O‐25% precursor) were only used (without storage) to compare any changes in performance.

**Figure 1:**
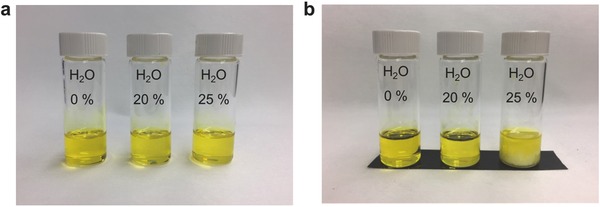
Photograph of perovskite (CH_3_NH_3_PbI_3−_ *_x_*Cl*_x_*) precursor solutions with various H_2_O concentrations. a) Freshly prepared precursor solutions after filtering and b) the same precursor solution after storage overnight. Note that only the 25% H_2_O solution appears cloudy after storage.

To study the influence of water containing precursors on the device performance, we prepared inverted structure perovskite solar cells with anhydrous precursor and H_2_O‐20% precursor. The freshly prepared H_2_O‐25% precursor was also used to fabricate devices prior to any precipitation to study the influence of higher concentration of water in the precursor. **Figure** **2**a shows the structure and cross‐section scanning electron microscopy (SEM) image of a device prepared with H_2_O‐20% precursor. A thin layer of poly(3,4‐ethylene‐dioxythiophene):polystyrenesulfonate (PEDOT:PSS) was first spin coated on the substrate. Perovskite layers were spin coated from a single precursor solution (one‐step method) and then dried using a vacuum‐assist method^30^, ^31^, ^32^, ^33^, ^34^ that resulted in the formation of smooth perovskite films. A 20 nm C_60_ layer was thermally evaporated on the perovskite film to aid in electron extraction. Finally, 7.5 nm of 2,9‐dimethyl‐4,7‐diphenyl‐1,10‐phenanthroline (BCP) and 180 nm of silver were thermally evaporated to make an Ohmic contact.

**Figure 2:**
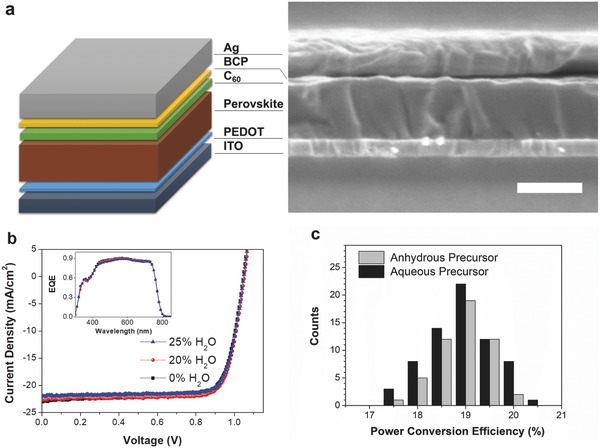
a) Device architecture and cross‐section SEM images of the perovskite solar cells prepared by H_2_O‐20% precursor. The scale bar is 300 nm. b) Current–voltage (*J*–*V*) curves of perovskite solar cells prepared by anhydrous‐ and aqueous‐containing precursor measured under 0.971‐sun illumination. The inset shows the corresponding EQE spectra of the perovskite solar cells. c) Histograms of PCE measured for 68 separate H_2_O‐20% precursor devices (black) and 51 separate anhydrous‐precursor (gray) devices.

The current–voltage (*J–V*) characteristics and external quantum efficiency (EQE) spectra of the devices are shown in Figure 2b. As can be seen from the *J–V* curve measured under standard AM1.5G illumination (with a spectral‐mismatch factor, *M*, of 0.971), all devices show good photovoltaic performance with PCE of up to 20.1%. These device performances are surprising as they do not exhibit any obvious decrease with water concentration as described in previous reports, which used only dilute concentrations of water in the precursor solution. The H_2_O‐20% precursor device shows nearly identical *J–V* and EQE data with devices made with the anhydrous‐precursor device. Additionally, the H_2_O‐25% precursor device also performs well, with a high FF of 79.5%, *V*_OC_ of 1.05 V, and a short‐circuit current density *(J*_SC_) of 21.9 mA cm^−2^, resulting in a PCE of 18.9%. Despite a slight reduction in *J*_SC_, the H_2_O‐25% precursor device still shows a comparable performance to the other two precursor devices. The result indicates the water content only limits the solubility of the precursors, but does not influence the device performance. To verify the reproducibility of the results, over 50 separate H_2_O‐20% precursor devices and anhydrous‐precursor devices were fabricated and tested. The histograms of the device performance characteristics are shown in Figure 2c (PCE) and Figure S1 in the Supporting Information (*J*_SC_, *V*_OC_, FF), and the average device parameters are summarized in Table S1 in the Supporting Information. The average PCE values of H_2_O‐20% precursor devices are slightly higher than the anhydrous‐precursor devices, but also show a slightly higher standard deviation. Other parameters are quite close and exhibit low variation. Both types of precursor devices show a high average FF of 0.78, which implies that the perovskite films have good charge collection and low defect density that contribute to device PCEs exceeding 18%. These results clearly suggest that there are no obvious changes in the device performance when replacing the anhydrous solvent with a large water fraction in the precursor solution.

To probe the influence of H_2_O in the precursor solution, the morphologies of the two perovskite films were studied. The SEM image of perovskite films prepared with the H_2_O‐20% precursor (**Figure** **3**b) shows a film that is smooth and continuous. Notably, the perovskite grain size is clearly larger than the anhydrous‐precursor film (Figure 3a) as can be seen in the SEM images and confirmed by the peak‐width reduction in the powder X‐ray diffraction (XRD) data in Figure 3c.^9^ This phenomenon is similar to that reported in previous research that only studied at the low water contents and found large voids at H_2_O content >2%.^28^ In our prepared perovskite films, pinholes were absent not only for anhydrous‐precursor films, but also for the H_2_O‐20% precursor‐based films which is primarily attributed to the vacuum‐assisted drying process.^30^, ^32^ Under vacuum, most of the solvent (DMF, dimethyl sulfoxide (DMSO), and water) can be extracted from the film, leading to the formation of crystalline perovskite grains followed by residual solvent evaporation. Since water prolongs the film drying time of DMF‐based perovskite precursor,^35^ it reduces the nucleation rate of perovskite grains, resulting in a larger grain size of the perovskite film. In addition, contact angle measurements (Figure S2, Supporting Information) indicate that the aqueous‐containing solvent shows lower wettability with the substrate than the anhydrous solvent, which can contribute to the formation of larger grain size of perovskite film for the aqueous‐containing precursor solution.^36^ Figure 3c shows the XRD pattern of perovskite films prepared by the anhydrous precursor and the H_2_O‐20% precursor, respectively. The XRD patterns show clear and sharp characteristic perovskite peaks for both films without any precursor (e.g., PbI_2_) peaks and with a single preferential (110) orientation. The H_2_O‐20% precursor‐based film shows the narrowest diffraction peaks (at the instrument resolution), which again indicates the H_2_O‐20% precursor can produce larger perovskite crystal grains (Figure S3, Supporting Information).

**Figure 3:**
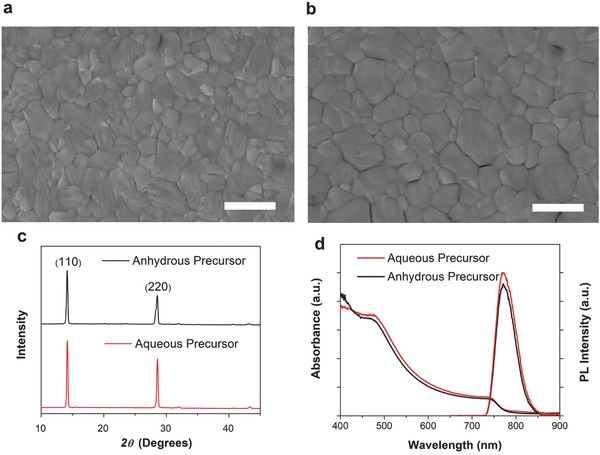
Top‐view SEM images of perovskite film prepared by anhydrous precursor a) and aqueous‐containing precursor b). The scale bar is 1 µm. c) Thin‐film XRD patterns of perovskite films. d) Absorbance spectra (measured in transmission mode) and PL spectra of perovskite films.

Figure 3d compares the absorbance and photoluminescence (PL) spectra for perovskite films. The slightly higher absorbance of H_2_O‐20% precursor‐based film in the range of 400–800 nm is consistent with a reduction in light scattering for a smoother and larger grained perovskite film. The steady‐state PL intensity is also slightly higher for H_2_O‐20% precursor‐based film. We infer that the H_2_O‐20% precursor can form a higher quality perovskite film with low defect density and few pinholes. Both the absorption and PL spectra results are consistent with the perovskite film characterization from SEM and XRD analyses.

Based on the above characterization, we found the aqueous‐containing precursor can form high‐quality perovskite films for efficient perovskite solar cells. More importantly, we found that by introducing this high fractional content of H_2_O in the precursor solutions, the perovskite film fabrication process does not exhibit any sensitivity with variations in humidity and can therefore be carried out in a range of conditions in air. As can be seen from **Figure** **4**a, when the relative humidity of the ambient air atmosphere changes from 16% ± 4% to 57% ± 3%, the device parameters only show a small fluctuation of about 5% (within the error of the measurements). While several reports have shown that perovskite films can be prepared under high humidity (≥50%) conditions,^37^, ^38^, ^39^, ^40^, ^41^, ^42^, ^43^ all of these reports resulted in limited device performance <16.61%. Our work indicates that the aqueous‐containing precursor enables a high tolerance for variation in the humidity of device fabrication even at the highest efficiencies, and therefore can enhance commercial processability by eliminating the need for nitrogen‐only or moisture‐free environments.

**Figure 4:**
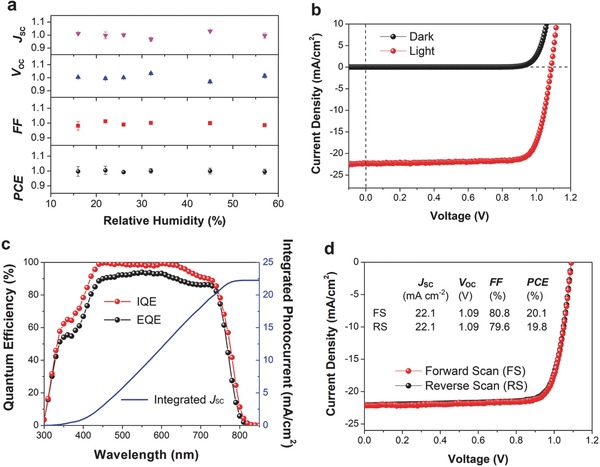
a) Normalized parameters of perovskite devices fabricated under various relative humidity conditions. Error bars represent one standard deviation from the mean. b) *J*–*V* curves in the dark and under 0.971‐sun illumination (with a spectral‐mismatch factor of *M* = 0.971) and c) the corresponding EQE spectra of the champion devices prepared by the H_2_O‐20% precursor. d) *J–V* curves of the champion H_2_O‐20% precursor device measured in reverse and forward bias.

The champion device prepared by the H_2_O‐20% precursor method resulted in a PCE of 20.1% (Figure 4b), with a *J*_SC_ of 22.3 mA cm^−2^, a *V*_OC_ of 1.09 V and an FF of 80.3%. As seen in the EQE spectrum from Figure 4c, the device shows EQE values above 80% from 420 to 740 nm. The integrated EQE of this device gives a *J*_SC_ of 22.2 mA cm^−2^, which is in good agreement with the measured value. The internal quantum efficiency (IQE) is also evaluated by measuring the device absorption via reflectivity measurements of the device (Figure 4c; Figure S4, Supporting Information). The IQE exceeds 95% in the range of 430–660 nm and indicates that the high quality of the as‐prepared perovskite film efficiently suppressing nearly all charge recombination loss. Furthermore, by measuring the *J–V* curve under forward and reverse bias, the device exhibits negligible hysteresis <1.5% (Figure 4d). We expect that the combination of aqueous‐containing precursor and vacuum‐assisted processing can enable high‐quality perovskite films prepared directly in air, which are obtained by improving the crystallization of the perovskite film. The large size of perovskite grains and the smooth surface are beneficial for reducing defect density, suppressing charge trapping, and yielding low hysteresis.^30^ We note that alternative approaches utilizing interface modification can be used to enhance the fast and barrier‐free electrons transfer process that can also provide efficient routes to eliminate the hysteresis in perovskite solar cells.^44^

Since the stability is an essential parameter for photovoltaic device viability, we further studied the initial stability of perovskite solar cells prepared with the H_2_O‐20% precursor and the anhydrous precursor in parallel. An organic solar cell was tested in parallel as the control cell during the duration of the perovskite test period (Figure S5, Supporting Information). **Figure** **5**a shows the stability test of H_2_O‐20% precursor device under continuous 1‐sun light irradiation (at 65 °C) for over 800 h. The PCE of the device exhibits a “burn‐in” in the first hour, due to the reductions in *J*_SC_ and FF that are common in many thin film photovoltaic devices. After this initial period, each device parameter changes more gradually, particularly the *V*_OC_, which maintains a value over 94% of the original value. It is notable that the device efficiency became stable after 100 h of test and as a result, the PCE maintains about 50% of the original value after 800 h of continuous light irradiation. Encouragingly, the aqueous‐containing precursor devices exhibit similar performance and lifetime with state‐of‐the‐art perovskite cells using TiO_2_ mesoporous scaffolds,^2^ indicating that the aqueous‐containing precursor process could become a commercially competitive technique. For the anhydrous‐precursor device (Figure 5b), while the FF increases by about 10%, the *V*_OC_ similarly shows an obvious reduction to less than 80% and offset the increase of FF. However, the *J*_SC_ has a significant drop to 1/4 of the original value by 200 h, finally yielding only a 12% of original PCE after 800 h test.

**Figure 5:**
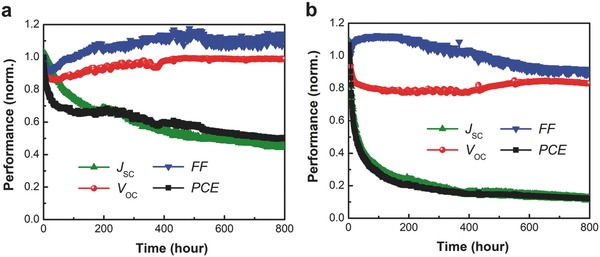
Initial device stability testing of perovskite solar cells for the a) H_2_O‐20% precursor devices and the b) anhydrous‐precursor devices measured under constant simulated solar illumination (100 mW cm^−2^) at 65 °C. The devices are encapsulated and illuminated with a sulfur‐plasma lamp, without UV‐filter. Four devices of each structure were tested and all showed similar behavior. Part of the degradation mechanism is tied to the degradation of the top Ag electrode (see Figure S7 in the Supporting Information).

To understand the mechanism of how the aqueous‐containing precursor improved the device stability, we performed degradation tests of perovskite films in ambient atmosphere (without packaging). The SEM images for perovskite films prepared by anhydrous‐ and aqueous‐containing precursor are show in **Figure** **6**. It is clear that the grain size of aqueous‐containing precursor film is larger than anhydrous‐precursor film. After being aged for 2 h in air under 65 °C and 45% relative humidity, the anhydrous‐precursor film shows significant degradation with a high number of pinholes and white spots. In contrast, the aqueous‐containing precursor only shows slight degradation with a smaller number of pinholes. The XRD characterizations indicate that the aged anhydrous‐precursor film exhibits more obvious degradation than aqueous‐containing precursor film, since the XRD pattern shows the emergence of a stronger diffraction peak for PbI_2_ at 12.64° (Figure S6, Supporting Information). We infer that the compact perovskite film with large‐size grains is responsible for the slower degradation with the aqueous‐containing precursor device. Additional work is needed, however, to identify the key component failure (perovskite, PEDOT:PSS, C_60_, or top Ag contact). Indeed, upon observation of the backside of devices after the stability test (Figure S7, Supporting Information), we found that the silver electrode became dark and cloudy, which indicates that the silver electrode is degrading and perhaps reacting with the perovskite layer.^16^, ^45^ The results also suggest that the perovskite device stability can be further improved by replacing the silver electrode with inert materials, such as copper‐ or carbon‐based electrodes.^8^, ^46^ Nonetheless, the instability of the perovskite absorber itself plays the important role on the instability of device in air. We observed that the PCE of unencapsulated device quickly drops to only 13% in 24 h (Figure S8, Supporting Information). In contrast, the reported aqueous‐containing precursor still shows shorter dark lifetime compared to the solvent‐free, cesium‐doped preparation method.^23^

**Figure 6:**
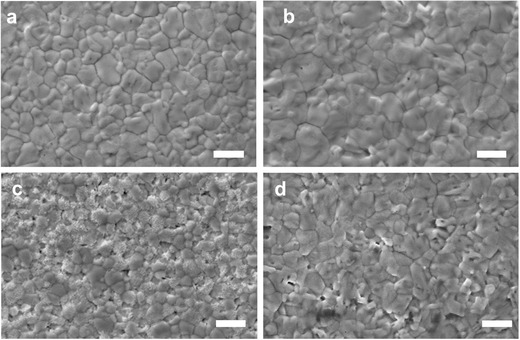
Top‐view SEM images of perovskite film before and after aging. Fresh a) and aged c) anhydrous‐precursor films; fresh b) and aged d) aqueous‐containing precursor films. The scale bar is 1 µm.

In summary, we have used an aqueous‐containing perovskite precursor with up to 25% water content to successfully fabricate highly efficient perovskite solar cells. This enabled the fabrication of perovskite cells with good reproducibility and high efficiency. This work highlights that an excess of water in perovskite precursor solution does not necessarily show a negative effect on the device performance and can actually enhance the performance and device stability while reducing hysteresis. Moreover, our work enables the fabrication of efficient perovskite devices with water‐containing solvents and high humidity conditions that lead to a reduction in moisture sensitivity for perovskite devices' preparation process and longer lifetime of devices. Since water is an economical and environmental friendly solvent, this research also provides a route to reduce the impact of the solvent system while easing the strict air‐free fabrication requirements.

## Experimental Section

*Materials and Precursor Preparation*: DMF (anhydrous, 99.8%, Aldrich), DMSO (anhydrous, 99.9%, Aldrich), PEDOT:PSS (Clevios PVP AI 4083, Heraeus, diluted to 10% by water for use), CH_3_NH_3_I (MAI, Lumtech.), PbI_2_(99%, Aldrich), PbCl_2_(98%, Aldrich), C_60_ (99.9%, MER Corporation) BCP (Lumtech.) were used as received.

To prepare the anhydrous perovskite precursor solution, MAI:PbI_2_:PbCl_2_ (191, 461, and 27.8 mg, respectively) were added in a mixed solvent of DMF and DMSO (0.637 and 0.213 mL, respectively). For 20% of aqueous perovskite precursor solution, MAI:PbI_2_:PbCl_2_ (191, 461, and 27.8 mg, respectively) were added in a mixed solvent of DMF and DMSO (0.510 and 0.170 mL, respectively), after the powder dissolved in the solvent with stirring, the deionized water (0.170 mL) was slowly added in the above solution under stirring. The solutions were then stirred for 1 h and filtered with 0.45 µm PTFE filters before use.

*Device Fabrication*: The PEDOT:PSS solution was spin coated onto precleaned ITO substrates at 6000 rpm for 10 s and then annealed at 110 °C for 5 min. The anhydrous‐ or aqueous‐containing perovskite precursor was spin coated on top of PEDOT:PSS film at 6000 rpm for 12 s, and then moved into a homemade vacuum chamber, evacuated to the mTorr range, and left in the chamber for 3 min. The substrates were then transferred to the hot plate and annealed at 80 °C for 10 min. The above operations were taken under ambient atmosphere with the relative humidity between 16% and 57%. The relative humidity value was measured by a humidity gauge (AcuRite, 5% error range) and recorded during cell fabrication from day‐to‐day variation. The substrates were then moved into the evaporation chamber for deposition of C_60_ (20 nm) and BCP (7.5 nm). Finally, a 180 nm thick silver layer was deposited by thermal evaporation at a base pressure of 3 × 10^−6^ Torr through a shadow mask with a final measured device area of 4.85 mm^2^.

*Measurement and Characterization*: The current density–voltage characteristics (*J*–*V* curves) were obtained using a Keithley 2420 source measurement unit under both dark and AM1.5 G solar simulation (xenon arc lamp with the spectral‐mismatch factor of 0.971.) where the light intensity was measured using an NREL‐calibrated Si reference cell with KG5 filter. Devices were scanned at a rate of 50 mV s^−1^. EQE measurements were performed using a QTH lamp with a calibrated Si detector, monochromator, chopper, and lock‐in amplifier. The IQE was evaluated by measuring the device absorption via reflectivity measurements of the overall device (*A* = 1 −*R*). XRD data were measured using Cu Kα (0.154 nm) emission with a Bruker D2 phaser. The *J–V*, EQE, absorption, PL, and XRD were measured on unencapsulated devices/samples in ambient air. A field‐emission scanning electron microscope (Carl Zeiss Auriga Dual Column FIB SEM) was used to acquire SEM images. Stability tests were conducted under constant illumination from a 500 W sulfur plasma lamp with 1‐sun intensity at 65 °C. Devices were held at maximum power point, and current–voltage characteristics were measured once every hour to extract performance parameters. Prior to lifetime testing, device substrates were edge sealed using UV‐cured epoxy in a nitrogen environment under cavity glass.

## Conflict of Interest

The authors declare no conflict of interest.

## Supporting information

Supplementary
