# Supplementary material for: Aqueous‐Containing Precursor Solutions for Efficient Perovskite Solar Cells
Source: Adv Sci (Weinh). 2017 Nov 10;5(1):1700484. doi: 10.1002/advs.201700484 (PMC5770683; doi:10.1002/advs.201700484)

Copyright WILEY-VCH Verlag GmbH & Co. KGaA, 69469 Weinheim, Germany, 2017.

ADVANCED SCIENCE
Open Access

Supporting Information

for *Adv. Sci.,* DOI: 10.1002/advs.201700484

Aqueous-Containing Precursor Solutions for Efficient Perovskite Solar Cells

*Dianyi Liu, Christopher J. Traverse, Pei Chen, Mark Elinski, Chenchen Yang, Lili Wang, Margaret Young, and Richard R. Lunt**

Copyright WILEY-VCH Verlag GmbH & Co. KGaA, 69469 Weinheim, Germany, 2016.

Supporting Information

Aqueous-Containing Precursor Solutions For Efficient Perovskite Solar Cells

*Dianyi Liu, Christopher J. Traverse, Pei Chen, Mark Elinski, Chenchen Yang, Lili Wang, Margaret Young and Richard R. Lunt**

Figure S1. Histograms of J_{SC}, V_{OC} and *FF* measured for 68 separate aqueous-precursor devices (black) and 51 separate anhydrous-precursor (grey) devices.

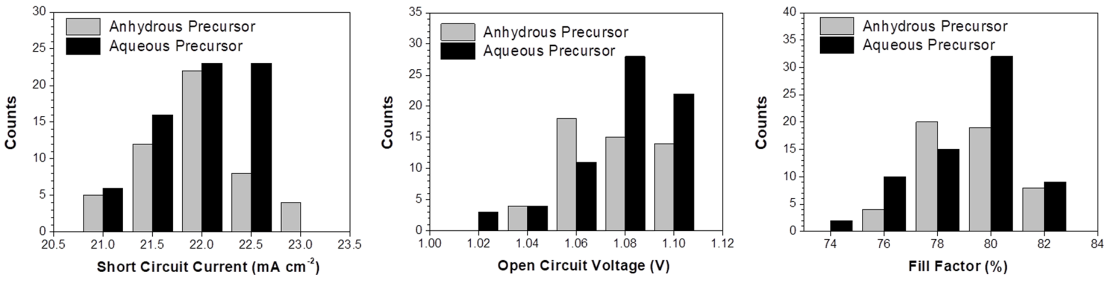

Table S1. Average device parameters for different precursor devices.

Precursor	# of devices	J_{SC} [mA cm^{-2}]	V_{OC} [V]	*FF* (%)	*PCE* [%]
Aqueous	68	21.7 ± 0.5	1.07 ± 0.02	78.1 ± 2.0	18.7 ± 0.7
Anhydrous	51	21.9 ± 0.5	1.07 ± 0.02	78.2 ± 1.6	18.6 ± 0.5

Figure S2. The contact angle of anhydrous solvent and aqueous-containing solvent on the ITO/PEDOT substrates. The anhydrous solvent quickly spreads once dropped onto the substrate and resulted in a contact angle close to 0°; the contact angle of the aqueous solvent on the substrate is 8°.

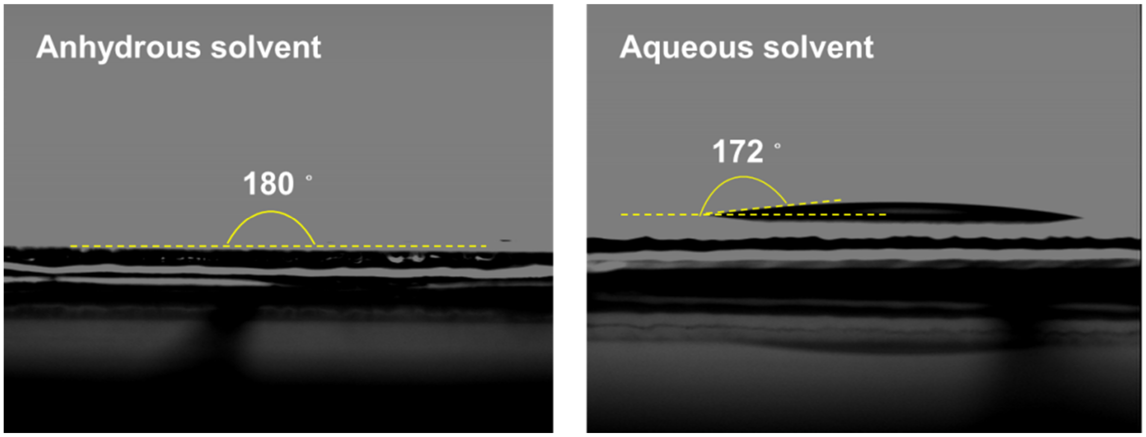

Figure S3. Calculation of grain size by XRD. (a) X-ray diffraction data peak profile fitting. For the anhydrous set, FWHMs are 0.2318° and 0.267°; for aqueous set are 0.194° and 0.218°. For the anhydrous set there are actually two close peaks that are deconvoluted around 2θ = 28° (28.257° and 28.543°). Only the FWHM for the dominant peak, which is in the same (*hh*0) family, is used for crystal size calculations. (b) Williamson-Hall plot for the anhydrous film gives a volume average grain size in the film normal as 754 Å. Note that the main peaks in both sets are predominately Gaussian in shape and the instrument broadening is subtracted accordingly. The FWHM for the aqueous film is essentially identical to that measured from NIST certified LaB_6 powder so the grain size is larger than the instrument resolution (≥ 2500 Å) for the aqueous set. β_{obs} is the observed integral breadth and β_{inst} is the instrumental integral breadth.

a

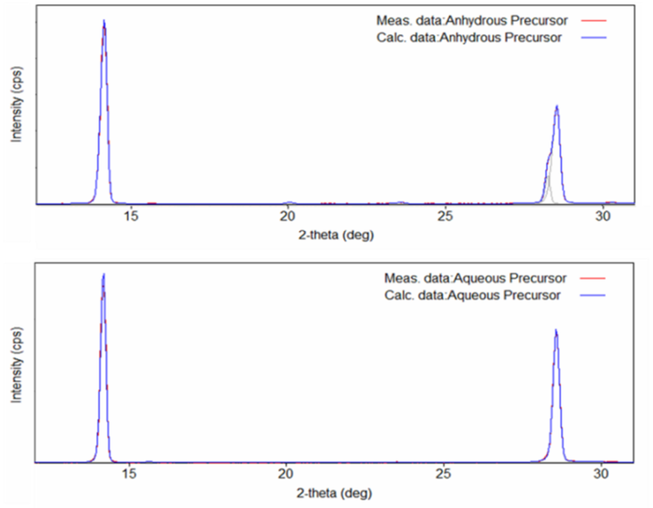

b

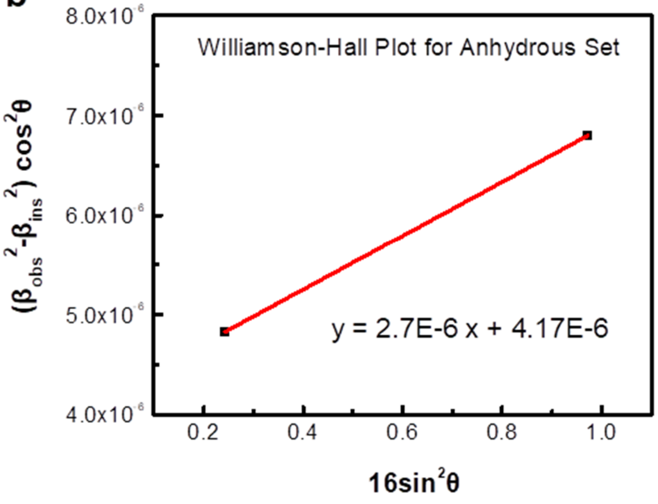

Figure S4. Absorption and quantum efficiency of H_2O-20% precursor device. Absorption spectrum and quantum efficiency of a H_2O-20% precursor based perovskite film and device, respectively. The absorption spectrum of the device is measured in the reflection mode as A = (1-R) and the IQE is calculated as EQE/A.

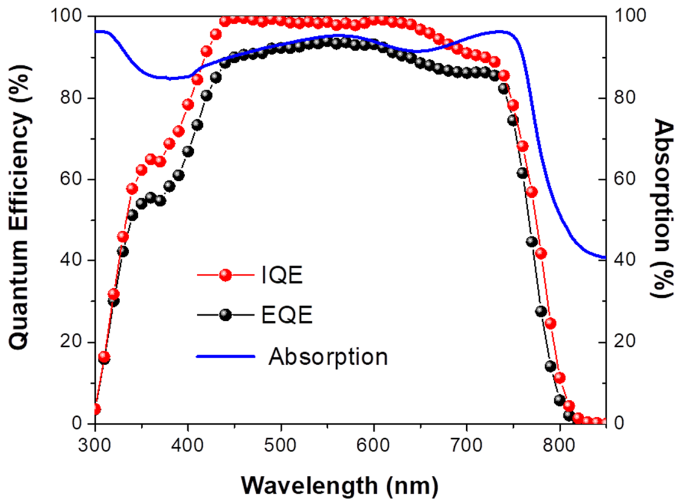

Figure S5. Stability test of an organic solar cell tested in parallel with the perovskite cells.

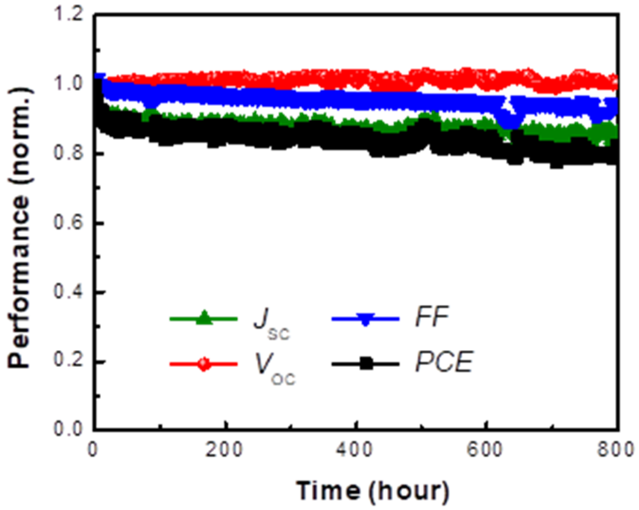

Figure S6. X-ray diffraction patterns of perovskite films before and after aging.

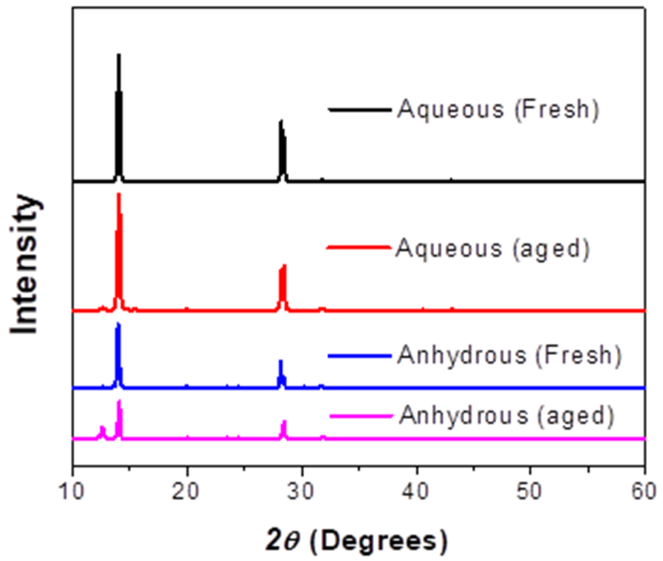

Figure S7. Photograph of perovskite solar cells. Photograph of perovskite solar cells (back side) after 100 hours of continuous illumination showing a cloudy formation on the Ag electrodes.

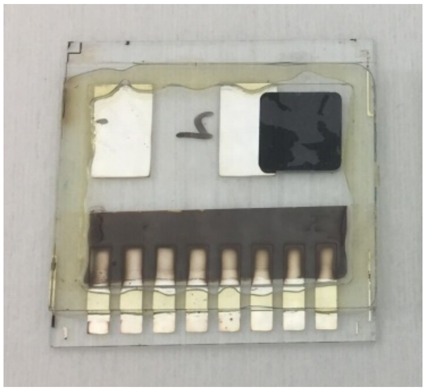

Figure S8. Stability test for an un-encapsulated aqueous-containing precursor device.

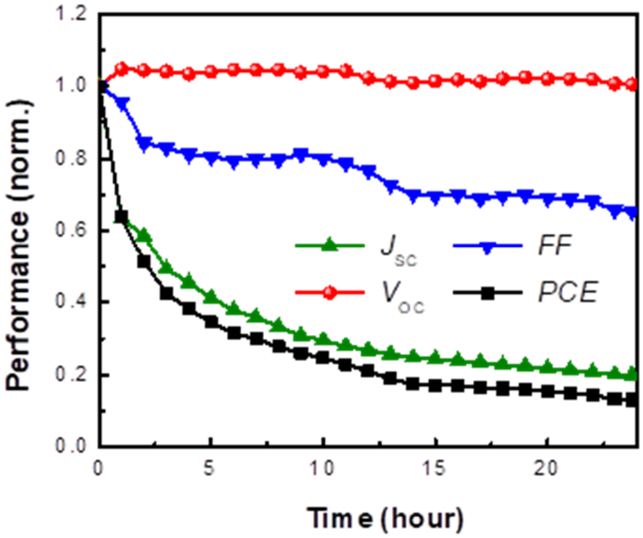

Supplement: Supplementary file 1 — Supplementary [file ADVS-5-na-s001.pdf]
